# Synthesis of Amorphous and Various Phase-Pure Nanoparticles of Nickel Phosphide with Uniform Sizes via a Trioctylphosphine-Mediated Pathway

**DOI:** 10.1021/acs.inorgchem.4c03334

**Published:** 2024-09-27

**Authors:** David Thompson, Adam S. Hoffman, Zachary R. Mansley, Sarah York, Feng Wang, Yimei Zhu, Simon R. Bare, Jingyi Chen

**Affiliations:** † Department of Chemistry and Biochemistry, University of Arkansas, Fayetteville, Arkansas 72701, United States; ‡ Stanford Synchrotron Radiation Lightsource, SLAC National Accelerator Laboratory, Menlo Park, California 94025, United States; § Interdisciplinary Science Department, Brookhaven National Laboratory, Upton, New York 11973, United States; ∥ Condensed Matter Physics and Materials Science Department, Brookhaven National Laboratory, Upton, New York 11973, United States

## Abstract

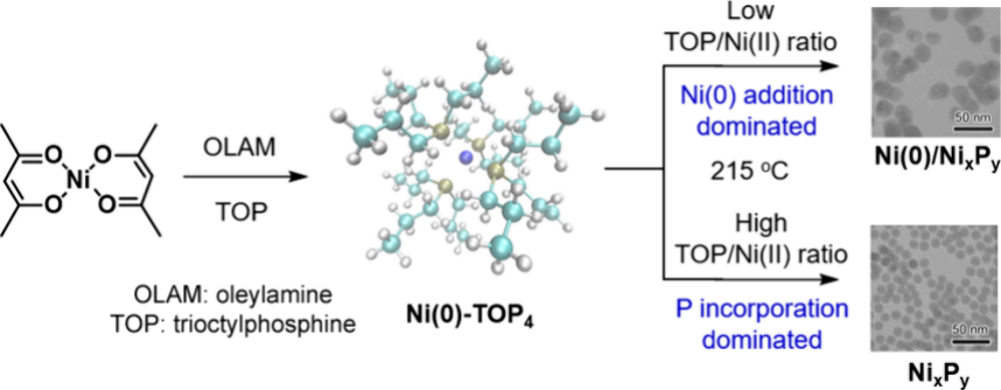

Nickel phosphides are of particular interest because they are highly active and stable catalysts for petroleum/biorefinery and hydrogen production. Despite their significant catalytic potential, synthesizing various phase-pure nickel phosphide nanoparticles of uniform size remains a challenge. In this work, we develop a robust trioctylphosphine (TOP)-mediated route to make highly uniform phase-pure Ni_12_P_5_, Ni_2_P, and Ni_5_P_4_ nanoparticles. The synthetic route forms amorphous Ni_70_P_30_ nanoparticle intermediates. The reactions can be stopped at the amorphous stage when amorphous particles are desired. The amount of P incorporation can be controlled by varying the ratio of TOP to Ni(II). The mechanism for composition control involves the competition of the kinetics of two processes: the addition of the reduced Ni and the incorporation of P into Ni. Uniform Ni_70_P_30_ amorphous nanoparticles can be generated at a high TOP-to-Ni(II) ratio, where the P incorporation kinetics is made to dominate. Ni_70_P_30_ can later be transformed into phase-pure Ni_12_P_5_, Ni_2_P, and Ni_5_P_4_ nanocrystals of uniform size. The transformation can be controlled precisely by modulating the temperature. A UV–vis study coupled with theoretical modeling reveals Ni(0)-TOP_*x*_ complexes along the synthetic path. This approach may be expanded to create other metal compounds, potentially enabling the synthesis of uniform nanoparticles of a greater variety.

## Abstract 2

The trioctylphosphine (TOP)-mediated synthesis of nickel phosphides involves a Ni(0)-TOP_4_ complex that facilitates the nucleation. The Ni(0) addition and P incorporation are the two competing processes during growth, which dictate the composition/phases of the product. The synthesis at a high TOP/Ni(II) ratio leads to the formation of uniform amorphous nickel phosphide nanoparticles, which can serve as an intermediate to synthesize phase-pure crystalline Ni_12_P_5_, Ni_2_P, and Ni_5_P_4_ nanoparticles with narrow size distributions.

## Introduction

Nickel phosphides are nonprecious metal compounds that are becoming a promising group of highly active and stable catalysts in the petroleum refining industry for hydrodesulfurization (HDS) and hydrodenitrogenation (HDN),^1,2^ in biorefinery for hydrodeoxygenation (HDO),^3^ and in energy conversion and storage.^4^ Their catalytic activity is strongly affected by the phase, size, and morphology of nickel phosphides in these applications such as HDS^5,6^ and overall water-splitting reactions.^7−9^ A number of methods have been developed for the synthesis of nickel phosphides involving the reactions between nickel salts and different phosphorus sources, including phosphates, hypophosphite, element phosphorus, and phosphines.^8^ Among these methods, the wet chemical approach using trioctylphosphine (TOP) as the phosphorus source is an effective means of synthesizing nickel phosphide nanoparticles. The general strategies often involve the thermal decomposition of metal–phosphine intermediates,^10^ thermal conversion of metals to phosphides,^11,12^ or a one-pot synthesis, combing the metal reduction and phosphorus incorporation.^13−18^ For example, Hyeon and co-workers reported that a series of metal–TOP intermediates could be thermally decomposed, leading to the formation of metal phosphide nanoparticles, including solid Ni_2_P particles.^10^ Schaak and Chiang demonstrated that the thermal conversion of metallic Ni to hollow Ni_2_P particles was due to the unequal diffusion rates of Ni and P, known as the Kirkendall effect.^11,12^ In the one-pot synthesis, the combination of the reduction of metal precursor and the P incorporation requires the presence of a reducing agent, where oleylamine (OLAM) is often used.

The control of the phase, size, and morphology (hollow versus solid) could be achieved in a one-pot synthesis by varying the reaction parameters, such as the P/Ni precursor ratio, temperature, time, and the amount of OLAM used. For example, Tracy and co-workers showed that at 240 °C, a 1:1 P/Ni ratio formed Ni particles, while a >9:1 P/Ni ratio yielded amorphous Ni_*x*_P_*y*_ particles.^14^ Further annealing of the Ni and the amorphous Ni_*x*_P_*y*_ particles individually generated a crystalline mixture of Ni_2_P and Ni_12_P_5_, but with different morphologies of hollow versus solid, respectively. Later, Brock and co-workers reported that phase-pure Ni_12_P_5_ and Ni_2_P could be modulated by the TOP quantity, reaction temperature, and heating time.^18^ In their study, the void size of the hollow particles could be tuned by the P/Ni precursor ratio and the amount of OLAM used in the synthesis. When phosphidation was performed in the reaction with a large excess of TOP and high temperatures, the products were P-rich phases of Ni_5_P_4_ and NiP_2_.^19^ However, the size of the resulting particles was large, typically in the range of 100–500 nm, which appeared to be aggregates consisting of 20–30 nm crystalline nanoparticles. Using a similar TOP-mediated synthesis, monodispersed particles of Ni_12_P_5_ (∼20 nm, hollow), Ni_2_P (∼10 nm, a mixture of hollow and solid), and Ni_5_P_4_ (∼600 nm, particle assemblies) were obtained by Liu and co-workers.^20^ A theoretical study showed that a clean Ni surface could initiate the P–C bond cleavage of TOP, facilitating the insertion of P to Ni to form nickel phosphides.^21^ The energy barriers of the entire process were relatively low, in agreement with the experimental findings, where the birth of nickel phosphides was at a relatively low temperature of 150 °C.^22^ Despite these advances of TOP-mediated synthesis, challenges still remain to have complete control over the size, shape, and phase of the nickel phosphide nanoparticles. Further decoupling of the reaction parameters is necessary to understand the reaction mechanism and thus achieve simultaneous control of the morphology and phase for the formation of nickel phosphide nanoparticles.

In this work, we decouple the reaction parameters of the TOP-mediated synthesis, propose a new reaction mechanism, and demonstrate a precise modulation strategy for the formation of phase-pure crystalline Ni_12_P_5_, Ni_2_P, and Ni_5_P_4_ nanoparticles with a uniform size. The investigation systematically increases the amount of TOP used in the synthesis of metallic Ni, which was formed by the thermal reduction of nickel(II) acetylacetonate (Ni(acac)_2_) by using OLAM, to study the TOP effects on the formation of Ni_*x*_P_*y*_. At a relatively low temperature, the P incorporation to the in situ formed metallic Ni results in amorphous phases. The amount of P incorporation mainly depends on the amount of TOP added to the reaction with little influence by the reaction time. Our study shows that the highest P incorporation is approximately 30% at the low reaction temperature, forming uniform Ni_70_P_30_ amorphous nanoparticles on the order of 10 nm with a standard deviation of less than 2.0 nm. These Ni_70_P_30_ amorphous nanoparticles can serve as reaction intermediates to the phase-pure crystalline Ni_12_P_5_, Ni_2_P, and Ni_5_P_4_ solid nanoparticles with little change in particle size. The key to success is through a two-step heating process, during which the uniform amorphous nanoparticles are formed at the intermediate temperature to facilitate the size and phase purity control of the final products. The as-synthesized nanoparticles can be immobilized to SiO_2_ support^6^ or metal foil^23^ and annealed at elevated temperatures before their use in catalysis. They can also be chemically treated through ligand exchange^24^ or covalent surface functionalization^25^ before deposition onto carbon support for electrocatalysis. We further employ X-ray absorption spectroscopy (XAS), including both X-ray absorption near-edge spectroscopy (XANES) and extended X-ray absorption fine structure analysis (EXAFS), to analyze the amorphous nanoparticle intermediates from the synthesis. The results, combined with transmission electron microscopy (TEM), X-ray diffraction (XRD), UV–vis spectroscopy, and density functional theory (DFT) calculations, allow us to reveal the Ni(0)-TOP_*x*_ involving the reaction mechanism and deepen the understanding of the TOP-mediated nickel phosphides at the nanoscale.

## Experimental Methods

### Chemicals and Materials

Nickel(II) 2,4-pentadionate (Ni(acac)_2_, 95%), tri-n-octylphosphine (TOP, 90%,), chloroform (CHCl_3_, 99.8%+), toluene, and nitric acid (65–70%, ≥99.999% (metals basis)) were purchased from Alfa Aesar. OLAM (70%), 1-octadecene (ODE, 90%), and acetone were obtained from Sigma-Aldrich. Ethanol (200 proof) was obtained from Koptic. All chemicals were used as received. Ultrapure water was used unless specified.

### Synthesis of Crystalline Ni

Crystalline Ni nanoparticles were synthesized by thermal decomposition of the nickel precursor under a reductive environment. In a typical synthesis, Ni(acac)_2_ (52.4 mg, 0.2 mmol) was added to 5 mL of OLAM in a three-neck round-bottom flask equipped with a Schlenk line under an argon flow. The mixture was then degassed for 10 min under argon. Next, the reaction mixture was heated to 215 °C and held at 215 °C for 15 min. After 15 min, the reaction was quenched by removing the reaction flask from the heating mantle. Once the solution mixture was cooled to 100 °C, the solution was transferred to a 50 mL centrifuge tube, mixed with 3 mL of toluene and 30 mL of ethanol, and centrifuged at 8000 rpm for 5 min. After centrifugation, the supernatant was decanted, and the pellet was purified twice using a 1:10 mixture of toluene and ethanol. The product was dispersed in 3 mL of toluene for future use.

### Synthesis of Amorphous Ni_*x*_P_*y*_ Nanoparticles

To synthesize the amorphous Ni_*x*_P_*y*_ nanoparticles, TOP was added to the synthesis of Ni nanoparticles as a source of phosphorus. Briefly, different amounts of TOP (0.05, 0.2, 0.5, or 1 mL) were added to a three-neck round-bottom flask containing Ni(acac)_2_ (52.4 mg, 0.2 mmol), 1 mL of OLAM, and 4 mL of ODE. The reaction was heated to 215 °C and held at 215 °C for various periods of time (10, 30, 60, and 120 min). After the specific time for the reaction to proceed, the reaction was quenched, and the resulting nanoparticles were purified using the same procedure as that for the synthesis of crystalline Ni nanoparticles. The product was further purified by acetone and then dispersed in 3 mL of toluene for future use.

### Synthesis of Crystalline Ni_2_P Nanoparticles

Crystalline Ni_2_P nanoparticles were synthesized by using the same procedure as that for the amorphous Ni_*x*_P_*y*_ except that an additional heating step at 315 °C was applied to the synthesis. Briefly, Ni(acac)_2_ (52.4 mg, 0.2 mmol), 1 mL of OLAM, and 4 mL of ODE were added to a three-neck round-bottom flask equipped with a Schlenk line under an argon gas flow. After the mixture was degassed for 10 min under argon, 1 mL of TOP was added to the flask. The reaction mixture was then heated to 215 °C and held at 215 °C for 1 h. After 1 h, the reaction was heated to 315 °C and held at 315 °C for 1 h before the reaction was quenched by removing the reaction flask from the heating mantle. The product collection and purification procedures are the same as those for the amorphous Ni_*x*_P_*y*_ nanoparticles.

### Synthesis of Crystalline Ni_12_P_5_ Nanoparticles

Crystalline Ni_12_P_5_ nanoparticles were synthesized by using the same procedure as that for crystalline Ni_2_P nanoparticles, except an additional heating step with the temperature held at 280 °C.

### Synthesis of Crystalline Ni_5_P_4_ Nanoparticles

The same procedure as that for the synthesis of crystalline Ni_2_P nanoparticles was applied for the synthesis of crystalline Ni_5_P_4_ nanoparticles, except that the amounts of TOP (1 mL), OLAM (5 mL), and no ODE were used, and the additional heating step was raised to 350 °C and held at 350 °C for 1 h.

### Transmission Electron Microscopy Characterization

For TEM sample preparation, each nanoparticle suspension was drop-cast onto a TEM grid and air-dried. Low-magnification TEM images were captured using a JEOL JEM-1011 microscope with an accelerating voltage of 100 kV. High-resolution TEM and high-angle annular dark-field (HAADF)-scanning transmission electron microscopy (STEM) images were acquired using a JEOL ARM200F microscope operated at an accelerating voltage of 200 kV and equipped with a cold-field emission gun and CEOS GmbH double C_s_ aberration correctors. The inner and outer collection angles for the HAADF images used were 68 and 280 mrad, respectively. The two-dimensional electron energy loss spectroscopy (EELS) mapping of the P L-edge was carried out using a Gatan GIF Continuum K3-IS System with the dual-EELS mode used for zero-loss energy-loss calibration. A power-law background subtraction was applied, and the data were processed using principal component analysis to reduce noise.

### X-ray Diffraction Characterization

To prepare the samples for XRD, each nanoparticle sample was precipitated by centrifugation, and the solid was dried under a nitrogen flow. The solid sample was placed on an XRD polymer loop that was mounted on a holder. XRD was performed using an X-ray diffractometer (Rigaku XtalLab Synergy) operated at 50 kV and 1 mA with Cu Kα radiation as the source.

### Inductively Coupled Plasma Mass Spectrometry Characterization

For inductively coupled plasma mass spectrometry (ICP-MS) sample preparation, 143 μL of concentrated HNO_3_ was added to each dry sample in a 2dr vial to digest the sample. The solution containing the digested sample was then diluted to 5 mL by using H_2_O to yield the final matrix concentration of 2% HNO_3_. The concentrations of Ni and P in each sample were obtained from ICP-MS (Thermo Fisher iCAP TQ). The linear ranges used for the calibration curve for Ni and P are 1–1000 and 50–1000 ppb, respectively.

### UV–Vis Spectroscopy Study

The reaction was carried out at the same conditions as the synthesis of amorphous Ni_*x*_P_*y*_ nanoparticles, with 1 mL of TOP used. During the course of the reaction, a 10 μL aliquot was sampled from the reaction and diluted in 2 mL of ODE in a quartz cuvette with a sealed cap for UV–vis measurements at different time points. The UV–vis spectra were recorded using a UV–vis spectrophotometer (Agilent Cary 50, Santa Clara, CA, USA), scanned from 200 to 800 nm at a scan rate of 10 nm per second.

The molecular structures were built using the GaussView graphical interface and optimized using ORCA,^26^ with density fitting DFT using Becke’s 3-parameter Lee–Yang–Parr (B3LYP) exchange–correlation functional.^27^ The Coulombic and exchange integrals were approximated with the RIJCOSX algorithm.^28^ Geometry optimization was performed with the def2-SVP^29^ basis set. However, a larger def2-TZVP basis set was used to compute the UV spectra with time-dependent DFT. The geometry optimization was performed in vacuum, assuming both singlet and triplet ground states. The singlet configuration was found to be more stable for all of the compounds reported. The UV spectra were computed with the singlet ground state in an implicit solvent of toluene approximated with the conductor-like polarizable continuum model.^29^ A total of 15 states were considered for computing the UV spectra.

### X-ray Absorption Spectroscopy Characterization

Each sample (∼60 μg/cm^2^, 0.5 cm diameter circle) was sandwiched between two pieces of Kapton tape. Ni K-edge XAS data were collected at beamline 4-1 of the Stanford Synchrotron Radiation Lightsource (SSRL) at SLAC National Accelerator Laboratory. These samples were placed in the beam path perpendicular to the incident X-ray beam. The transmission signal was collected for the Ni K-edge (8333.0 eV). Energy calibration was achieved by simultaneously scanning a Ni foil with each sample. Data calibration and analysis were carried out using the Demeter software package.^30^ The absorption edge energy for the Ni K-edge was calibrated to 8333.0 eV, and the EXAFS models were optimized in *R*-space using *k*^1^, *k*^2^, and *k*^3^ weightings, obeying the Nyquist criterion. The amplitude reduction factor (*S*_0_^2^) was determined by modeling the EXAFS spectra of the pure metallic Ni to be 0.75. The following Fourier transform (FT) parameters were chosen: *k*_min_ = 2.5 Å^–1^, *k*_max_ = 14 Å^–1^ for Ni_98_P_2_ and Ni_95_P_5_; *k*_min_ = 2.5 Å^–1^, *k*_max_ = 13 Å^–1^ for Ni_90_P_10_, Ni_85_P_15_, and Ni_80_P_20_; *k*_min_ = 2.5 Å^–1^, *k*_max_ = 10 Å^–1^ for Ni_75_P_25_ and Ni_70_P_30_; *dk* = 1, *r*_min_ = 1 Å, *r*_max_ = 3.0 Å, and d*r* = 0 with *k-*spline at 15 Å^–1^ were used for all of the samples. A simultaneous fitting approach using scattering paths chosen from metallic Ni^31^ (Ni–Ni) and Ni_2_P^32^ (Ni–Ni and Ni–P) was used to model Ni EXAFS spectra for all of the samples, where Ni_98_P_2_ and Ni_95_P_5_ were only fitted with metallic Ni–Ni, while others were fitted with all three paths. In this approach, for the Ni–Ni path of metallic Ni, the samples shared the same Δ*E*, σ^*2*^, and R. The CN was fitted individually. Similarly, this setting was also applied for the Ni–Ni and Ni–P paths from Ni_2_P with the exception of Δ*E*, which was shared for both paths. A total of 25 parameters (2 Δ*E*, 3 σ^2^, 3 R, and 17 CN) were fitted in the EXAFS modeling.

## Results and Discussion

**Figure 1:**
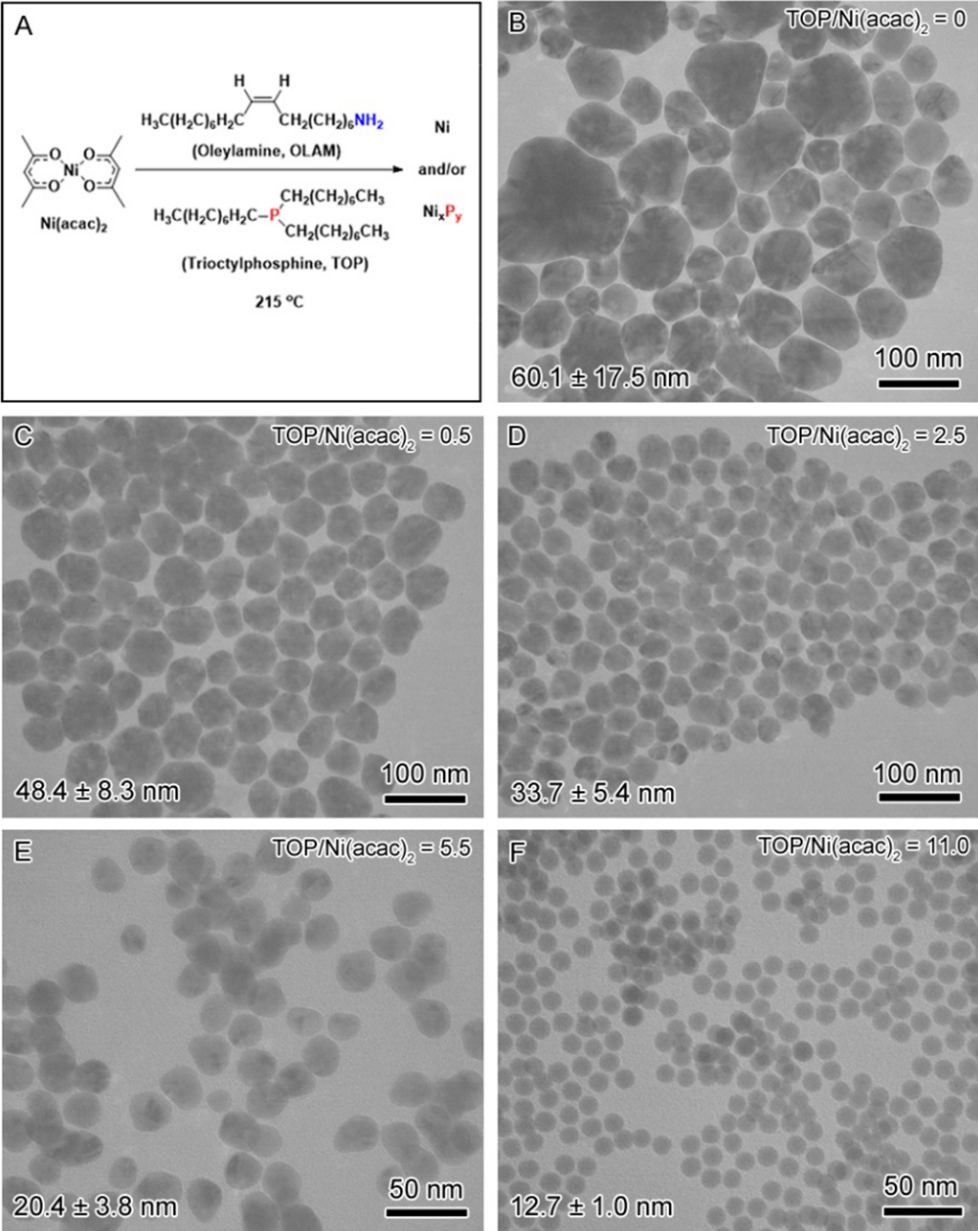
(A) Reaction scheme of the chemical approach to amorphous Ni_*x*_P_*y*_ via phosphorus insertion. (B) TEM image of Ni nanoparticles synthesized at 215 °C for 60 min via reduction of Ni(acac)_2_ in OLAM without the presence of TOP. (C–F) TEM images of the products from the reactions under the same conditions at 215 °C for 60 min but varying the molar ratio of TOP/Ni(acac)_2_: (C) 0.5; (D) 2.5; (E) 5.5; and (F) 11.0.

Nickel phosphides were synthesized through the incorporation of P obtained from TOP to the Ni generated in situ from the thermal reduction of Ni(acac)_2_ by OLAM. The reaction scheme is illustrated in Figure 1A. In the absence of TOP, Ni(acac)_2_ could be reduced by OLAM to Ni at 215 °C. The reaction mixture started as an opaque light blue color due to insoluble Ni(acac)_2_ suspended in OLAM at room temperature. Upon heating to 40 °C, Ni(acac)_2_ is fully dissolved under magnetic stirring, and the mixture turns translucent light green. By heating continuously to 215 °C, the reaction mixture gradually becomes translucent dark green and remains dark green until 15 min at 215 °C, at which point the mixture turns to opaque dark black, indicating the formation of Ni nanoparticles. The observation matches with the previous study of the reduction of Ni(acac)_2_ by OLAM.^33^ The mechanism for the reaction was proposed to undergo a two-electron chemical reduction route,^33^ as opposed to a thermolysis^34^ or a radical reaction.^35^

In the presence of TOP, Ni_*x*_P_*y*_ is formed by replacing Ni on the Ni surface.^22^ At a TOP/Ni(acac)_2_ ratio of 0.5, the reaction mixture turns opaque dark black at the time similar to that of the Ni synthesis—15 min after the reaction mixture is heated to 215 °C. As the TOP/Ni(acac)_2_ ratio increases, the temperature at which the reaction mixture turns opaque dark black decreases, occurring at 215, 205, and 185 °C, for ratios of 2.5, 5.5, and 11.0, respectively. This suggests that the increased amount of TOP in the reaction lowers the energy barrier for the nanoparticles to form. After the reaction temperature was ramped to 215 °C, all of the reactions proceeded at 215 °C for 60 min. Figure 1B–F displays the TEM characterization of the products from the thermal reduction of Ni in the absence and in the presence of TOP indicated by the TOP/Ni(acac)_2_ ratio of 0, 0.5, 2.5, 5.5, and 11.0. The corresponding atomic compositions of the products were measured by ICP-MS to be pure Ni, Ni_99_P_1_, Ni_95_P_5_, Ni_86_P_14_, and Ni_72_P_28_, respectively. As shown in the TEM images, the size of the particles decreases from 60.1 ± 17.5 nm (pure Ni) to 48.4 ± 8.3 nm (Ni_99_P_1_), 33.7 ± 5.4 nm (Ni_95_P_5_), 20.4 ± 3.8 nm (Ni_86_P_14_), and 12.7 ± 1.0 nm (Ni_72_P_28_). The size distribution becomes narrower, and the morphology changes from an irregular to a spherical shape as the P content increases in these samples. In other words, an increase of the TOP/Ni(acac)_2_ ratio in the synthesis facilitates the formation of uniform small solid nanoparticles with a higher P content.

**Figure 2:**
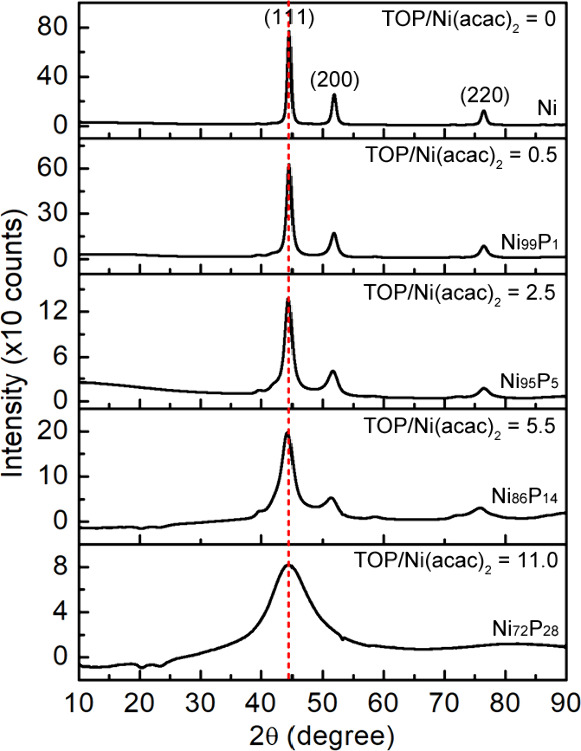
XRD of the samples that are depicted in Figure 1. The degree of crystallinity decreases with an increased ratio of TOP/Ni(acac)_2_ in the synthesis from the top to the bottom corresponding to the samples (Figure 1B–F). The pure Ni is indexed to an *fcc* crystal structure of Ni (ICDD-JCPDS card no. 04-0850). The compositions are the results obtained from ICP-MS.

To determine the corresponding crystalline phases, the XRD patterns of these samples were obtained, as shown in Figure 2. The XRD pattern of the pure Ni sample exhibits peaks at 44.5°, 51.9°, and 76.5° that can be indexed to the (111), (200), and (220) crystallographic planes of a face-centered cubic (*fcc*) structure of metallic Ni. The XRD patterns of these samples for Ni_99_P_1_, Ni_95_P_5_, and Ni_86_P_14_ are similar to those of the metallic Ni, as indicated by the presence of three major peaks at 44.5°, 51.9°, and 76.5°, but the peaks become broader as the P content increases. For Ni_72_P_28_, the broadening causes the convolution of the first two peaks of 44.5° and 51.9° and the disappearance of the weak peak of 76.5°, leaving only the peak at 44.5° in its XRD pattern. The peak broadening could be attributed to the reduced size and the reduced crystallinity of the particles due to the presence of P.

**Figure 3:**
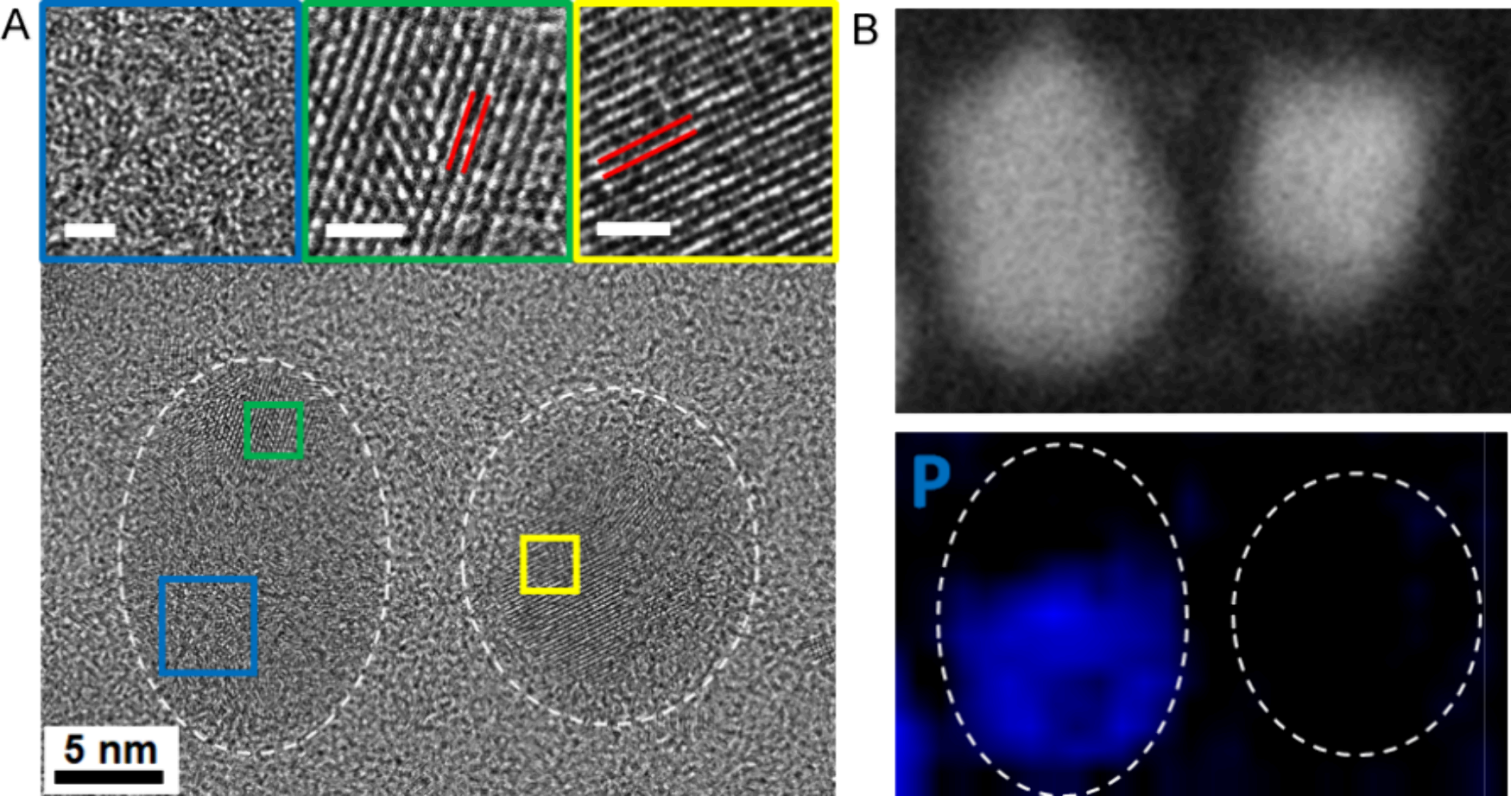
(A) HRTEM image of two nanoparticles from the Ni_85_P_15_ sample, which are circled in white, indicating a mixture of crystalline and amorphous domains. Magnified colored insets corresponding to the colored boxes on the image reveal an amorphous structure in the bottom half of the left-hand particle and crystalline domains in the top of the left-hand particle and in the right-hand particle (inset scale bars are 1 nm). In both cases, the lattice spacing indicated with red lines corresponds to Ni {111} (0.21 nm). (B) HAADF-STEM image and the EELS map showing phosphorus is mainly located within the amorphous regions of the nanoparticles.

We employed high-resolution TEM (HRTEM) and EELS mapping to analyze the phases at the single particle level. The TEM image in Figure 3A displays two particles in the Ni_85_P_15_ sample: one that is made up of an amorphous domain and a crystalline *fcc* Ni domain, while the other consists of crystalline *fcc* Ni. The EELS mapping in Figure 3B indicates that P is present only in the amorphous domain of the particles. The coexistence of crystalline Ni and amorphous Ni_*x*_P_*y*_ in a sample suggests that the addition of the reduced Ni and the incorporation of P to Ni simultaneously occur during the reaction. However, the rate of each process depends on the ratio of TOP/Ni(acac)_2_. When the ratio of TOP/Ni(acac)_2_ is small (e.g., ≤2.5), the addition of the reduced Ni dominates. At a ratio of TOP/Ni(acac)_2_ = 5.5, the rates of the reduced Ni addition and the P incorporation are equal, resulting in a heterogeneous mixture of pure Ni and Ni/Ni_*x*_P_*y*_ phase-segregated nanoparticles. When the ratio of TOP/Ni(acac)_2_ is large (e.g., = 11), the P incorporation outcompetes the addition of reduced Ni, resulting in amorphous Ni_*x*_P_*y*_ nanoparticles that lack long-range order. However, this cannot rule out the presence of very tiny crystallite Ni in the amorphous Ni_*x*_P_*y*_ nanoparticles.

**Figure 4:**
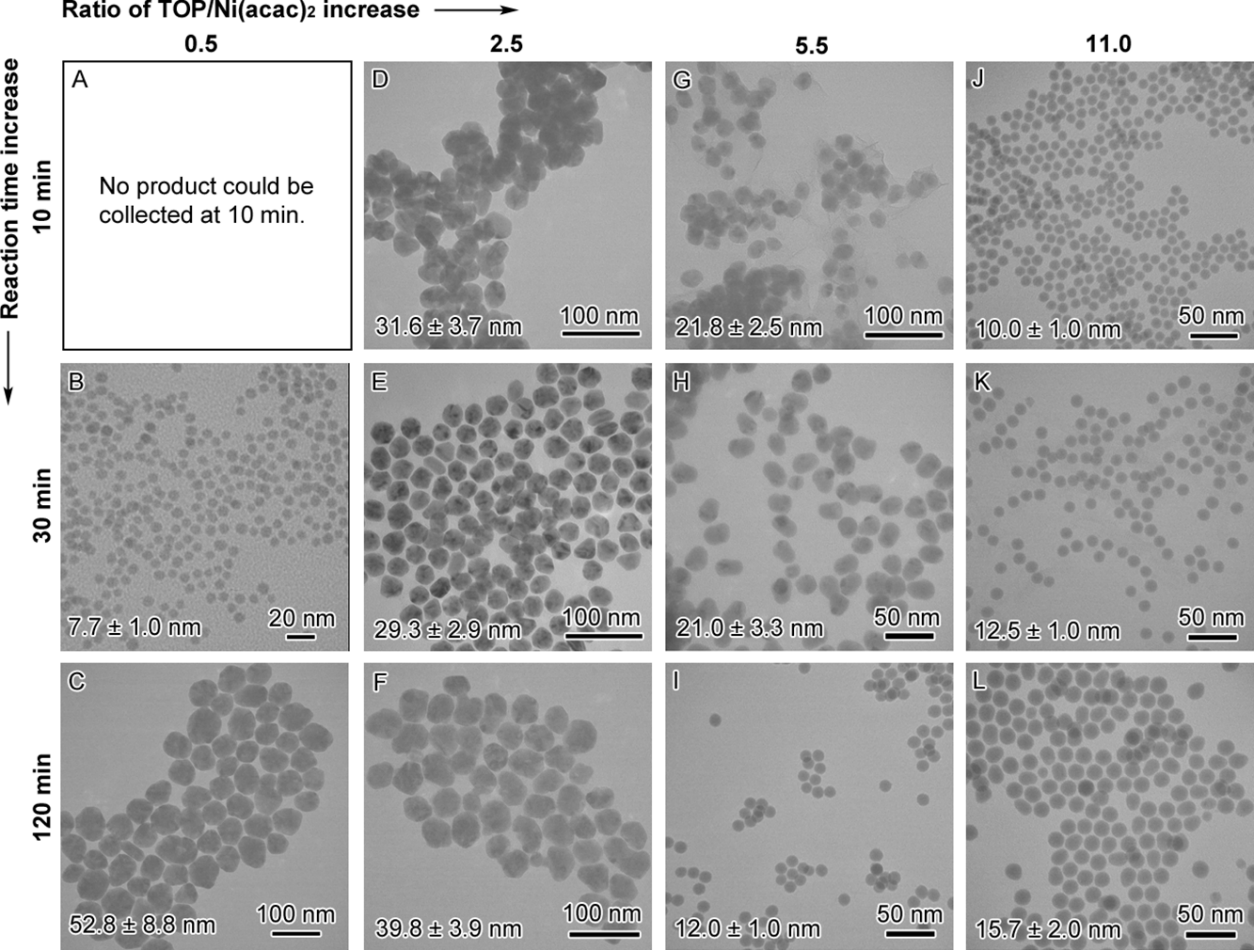
TEM images of the products from the TOP/Ni(acac)_2_ ratio and reaction time-dependent study under the same conditions at 215 °C: (A) 0.5, 10 min; (B) 0.5, 30 min; (C) 0.5, 120 min; (D) 2.5, 10 min; (E) 2.5, 30 min; (F) 2.5, 120 min; (G) 5.5, 10 min; (H) 5.5, 30 min; (I) 5.5, 120 min; (J) 11.0, 10 min; (K) 11.0, 30 min; and (L) 11.0, 120 min.

**Figure 5:**
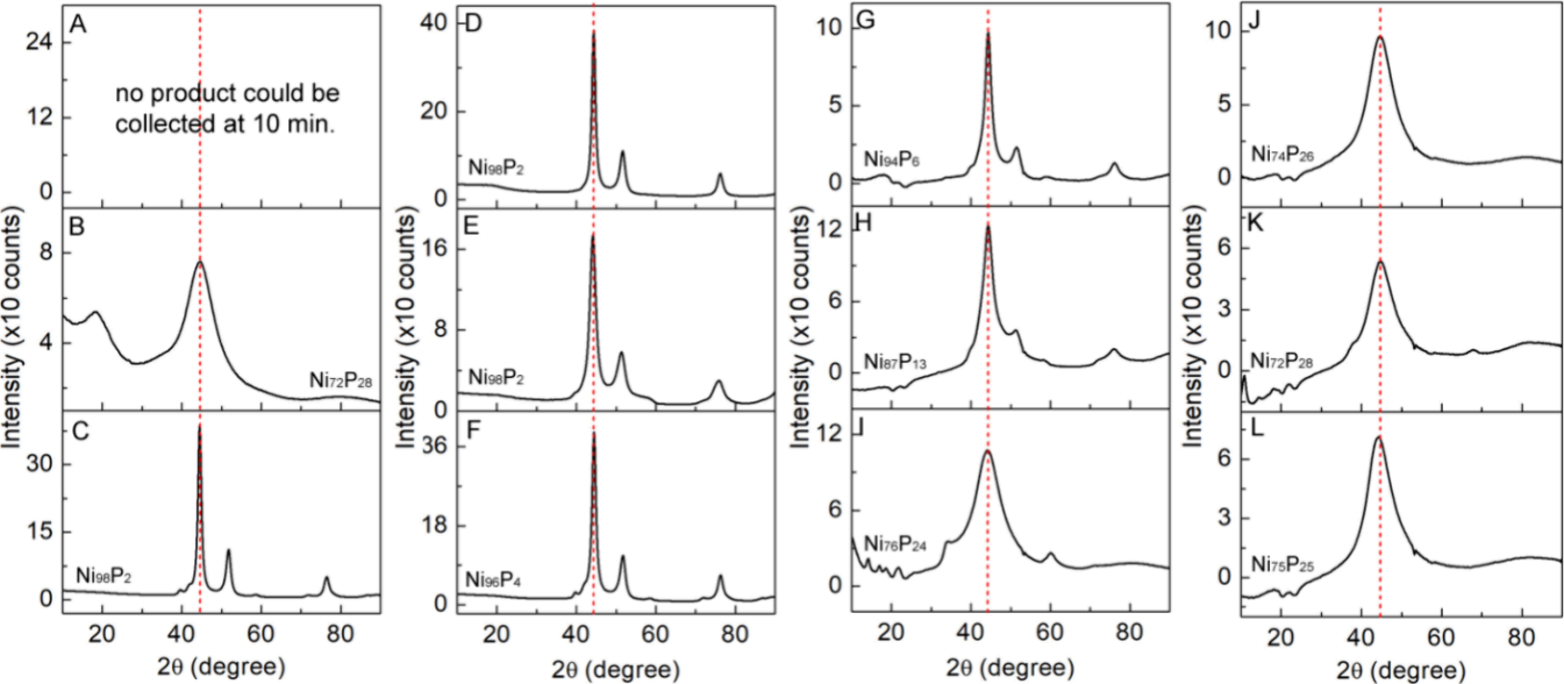
XRD of the corresponding samples that are shown in Figure 4. (A–L) Compositions are the results obtained from ICP-MS. The dashed line in red is the peak position corresponding to the (111) plane of the Ni XRD patterns.

It appears that the competing pair of reactions, the addition of reduced Ni and the replacement of Ni by P, affects the synthesis product. We further investigated the effects of reaction time and the ratio of TOP/Ni(acac)_2_ on the morphology and composition of the products. The results of TEM and XRD are shown in Figures 4 and 5, respectively. The ratios of Ni to P for the samples obtained from ICP-MS analysis are listed in Table S1. The particles with an average size of >20 nm have a set of peaks in their XRD patterns that can be indexed to metallic Ni. In contrast, the particles close to 10 nm are amorphous in nature with only one peak at 44.5° and their composition is very near Ni_70_P_30_. For comparison, we included the particle size measured from TEM and the Ni/P atomic ratio derived from ICP-MS results in Table S2.

From these results, we note some general aspects that affect the outcome of the synthesis. At a low TOP/Ni(acac)_2_ ratio of 2.5, the products are mainly crystalline Ni particles with less than 5% P, whereas at a high TOP/Ni(acac)_2_ ratio of 11.0, the products are largely amorphous small Ni_*x*_P_*y*_ particles with ∼25% P, regardless of the reaction time. At a moderate TOP/Ni(acac)_2_ ratio of 5.5, the products evolve from large crystalline Ni particles with 6% P content at 10 min to the same-sized crystalline Ni particles containing 13% P content at 30 min and finally small amorphous Ni_*x*_P_*y*_ particles with 24% P content at 120 min. However, when TOP/Ni(acac)_2_ is reduced to 0.5, there is no product that could be isolated for characterization at 10 min, but we were able to obtain small amorphous particles with 28% P at 30 min and large crystalline Ni particles with 2% P at 120 min. These observations suggest that the presence of Ni atoms facilitates the cleavage of P from TOP to form Ni–P at the initial stage of the synthesis. The formation of Ni_*x*_P_*y*_, in turn, promotes the reduction of Ni(II) to Ni. As the synthesis proceeds, if there is insufficient TOP in the reaction, the addition of reduced Ni will dominate the replacement of Ni by P, resulting in the crystalline Ni particles with only a small amount of P incorporation. However, if sufficient TOP is in the reaction, P will replace Ni to form small amorphous Ni_*x*_P_*y*_ nanoparticles with a higher percentage of P content. As the particles become smaller, the peak of the XRD pattern begins to be broad; however, there were no new sets of peaks formed that could be indexed to nickel phosphide crystalline phases. A further increase of the TOP/Ni(acac)_2_ ratio to 56 and the maximum 66 (used 6 mL of TOP alone) reduced the size of particles to 5.0 ± 1.0 and 3.8 ± 0.6 nm, respectively. Nonetheless, there were little changes to their composition (i.e., 69/31 Ni/P ratio or Ni_69_P_31_) and XRD patterns (i.e., one broad peak at 44.5°). The results are included in Figure S4. Therefore, we conclude that the nickel phosphides formed under these reaction conditions were amorphous. The results agree with the previously reported literature.^16,18^

**Figure 6:**
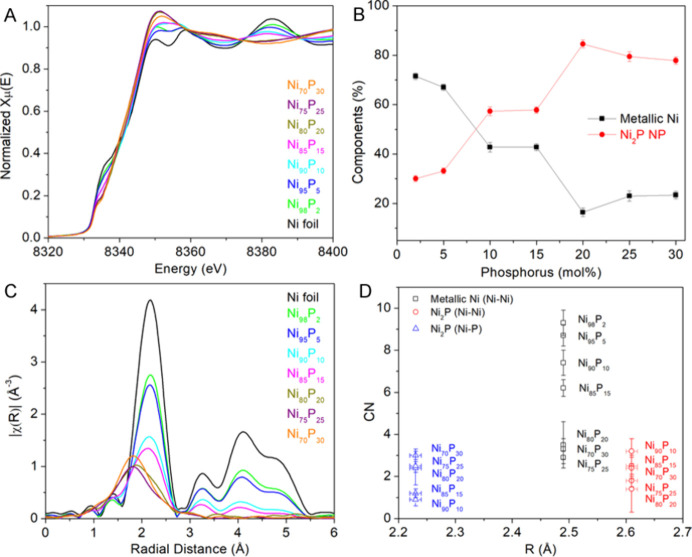
Ni K-edge XAS data of the Ni_*x*_P_*y*_ nanoparticles with different compositions: (A) Ni K-edge XANES spectra; (B) plot of percent distribution of metallic Ni and Ni_2_P obtained by LCF analysis; (C) Ni K-edge *k*^2^-weighted magnitude of the FT EXAFS spectra; and (D) Ni coordination number (CN) vs bond length (*R*).

In order to gain additional insight into the structure of Ni nanoparticles with P incorporation,^36,37^ we employed XAS to obtain local structural information around Ni in these samples. A series of Ni_*x*_P_*y*_ samples with the P content increasing from 2 to 30% measured by ICP-MS was chosen for the XAS study. Figure 6A displays a comparison of the Ni K-edge XANES spectra of the samples. The weak pre-edge feature arises from electric quadrupole transition from 1s → 3d of Ni.^38^ The intensity of this pre-edge feature at 8335.0 eV decreases with an increased P content from 0 to 15%. The pre-edge intensity can reflect the ratio of unoccupied 3d orbitals.^38,39^ The decrease in intensity corresponds to the increase of electron density of Ni due to the electron transfer from P atoms to Ni atoms for P content less than 15%, which agrees with the previous XAS study on the amorphous Ni_*x*_P_*y*_ alloys.^39^ As the P content increases in the range of 20–30%, the pre-edge peak shifts to 8334.0 eV. The small variation in peak intensity could be attributed to the coordination number and geometry.^38^ The XANES of Ni_*x*_P_*y*_ (*y* ≤ 15) closely resembles that of metallic Ni. As y increases to 20–30%, the shape of the near edge changes from two peaks with a valley to a single peak, which slightly shifts toward higher energy as the P content increases, an indication of electron transfer from Ni to P, resulting in an increase in the oxidation state of Ni. The analysis of HRTEM and EELS mapping allows us to identify that these nanoparticle samples may contain two components—metallic nickel and nickel phosphide. We, therefore, performed linear combination fitting (LCF) on the XANES data of these nanoparticle samples using the XANES data of metallic Ni and bulk crystalline Ni_2_P, as shown in Figures 6B and S1. As the amount of P increases, the metallic Ni component decreases; however, the relationship is not linear. The Ni and P percentages for each sample derived from the LCF results are listed in Table S3_._ Compared to the ICP-MS results, there is about a 5% discrepancy, which overestimates the P content at low concentrations but underestimates it at high concentrations.

We further performed modeling of the Ni K-edge EXAFS data to understand the coordination environment of Ni, including the bond length (*R*) and coordination number (CN) in these amorphous samples. Figure 6C displays the Ni K-edge *k*^2^-weighted magnitude of the FT EXAFS spectra. Compared to the Ni foil, changes can be seen for the peak resulting from the first scattering path of these samples. For *P* < 10%, the position and shape of the peak align with those of the Ni foil, but the intensity decreases by one-third. As the P content increases to 15%, the position of the first peak remains similar, while the intensity further decreases to one-third of the foil and the peak becomes broadened. With an incorporation of P to Ni of 20–30%, the position of the first peak shifts to a shorter distance and the shape of the peak is broader. In order to determine the number of phases present in these samples, we applied the continuous Cauchy wavelet transform (CCWT) tool,^40−43^ as shown in Figure S2. The 2 and 5% P-incorporated samples exhibit patterns similar to those of the Ni foil, which contains mainly one component in the first shell. We thus modeled these two samples with only the scattering path (Ni–Ni, *R* = 2.49 Å, CN = 12) from metallic Ni.^31^ At 10% P, an additional component is visible in the CCWT, and the intensity of this component becomes stronger as the percentage of P increases. Based on the HRTEM and EELS mapping results, we attribute this to the presence of amorphous nickel phosphides. Therefore, the EXAFS spectra of the samples with *P* ≥ 10% were modeled with both a Ni–Ni path from metallic Ni^31^ and a Ni–P path (*R* = 2.21 Å, CN = 2) and a Ni–Ni path (*R* = 2.61 Å, CN = 4) from Ni_2_P.^32^

The modeling results are listed in Table S4 and plotted in Figures 6D and S3. The CNs of metallic Ni are 9.3 ± 0.6 and 8.7 ± 0.5 at 2 and 5% P incorporated into the nanoparticles, respectively. The reduction of the CN from the bulk value (12) is likely due to P incorporation. For comparison, we also fitted these two samples using the paths from both phases. The CNs for Ni–P are 0.4 ± 0.3 and 0.3 ± 0.2 for Ni_98_P_2_ and Ni_95_P_5_, close to the calculated values of 0.4 and 0.6, assuming a homogeneous mixture and the P substitution of Ni in a *fcc* metallic phase.^44^ The result is also similar to the previously reported value for Ni_95_P_5_ (i.e., 0.6).^36^ When the percentage of P incorporation increases, the CN of metallic Ni continues to decrease to 7.4 ± 0.6, 6.2 ± 0.4, and 3.5 ± 1.1, for 10, 15, and 20% P incorporation, respectively, and then levels off at around 3 for 25 and 30% P incorporation. Meanwhile, the CN for Ni–P increases from 0.9 ± 0.3 to 3.0 ± 0.3 as the amount of P increases in the nanoparticles. Interestingly, the CN for Ni–Ni from Ni_2_P first decreases from 3.2 ± 0.6 at 10% P to 1.4 ± 1.1 at 20% P and then increases back up to 2.4 ± 0.5 at 30% P. It is worth noting that the Ni_90_P_10_, Ni_85_P_15_, and Ni_80_P_20_ nanoparticles were synthesized using a TOP/Ni(acac)_2_ ratio of 5.5, with increased reaction times from 10, 30, and 120 min, while the Ni_75_P_25_ and Ni_70_P_30_ nanoparticles were prepared using the TOP/Ni(acac)_2_ ratio of 11 with 10 and 60 min, respectively. With an increased reaction time, more P was incorporated into the nanoparticles in both cases. The rate of the P incorporation increases with an increased ratio of TOP/Ni(acac)_2_.

**Figure 7:**
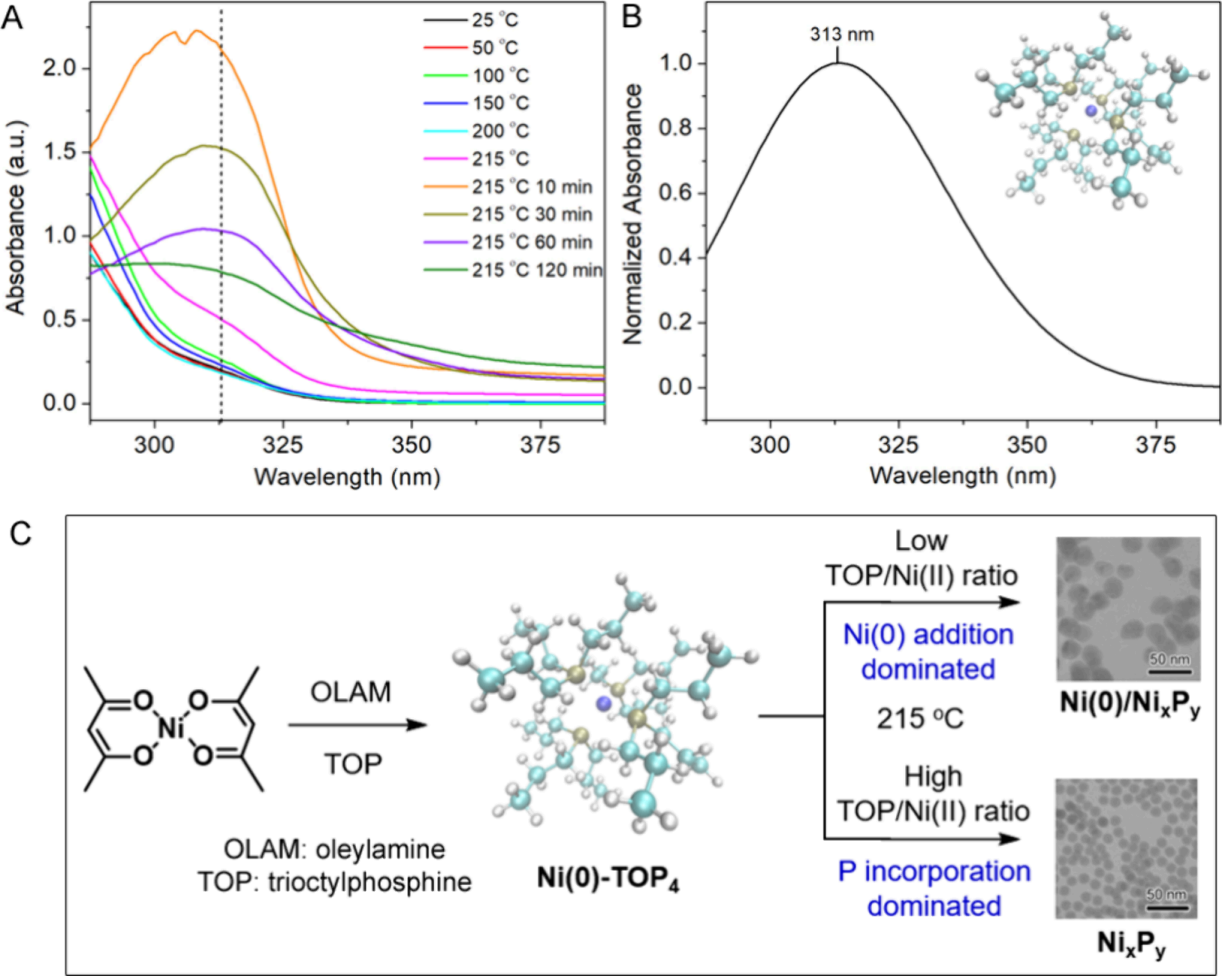
(A) UV–vis spectra of the aliquots taken from the reaction for the synthesis of the amorphous Ni_*x*_P_*y*_ with 1 mL of TOP used; (B) calculated UV–vis spectrum (λ_max_ = 313 nm) of a tetrahedral Ni(0)-TOP_4_ complex shown in the inset; and (C) schematic illustration of the reaction mechanism for the TOP-mediated synthesis.

To further understand the reaction mechanism, the reaction with a TOP/Ni(acac)_2_ ratio of 11 was monitored by UV–vis spectroscopy. Small aliquots (10 μL) were taken during the course of the synthesis and diluted in 2 mL of solvent (i.e., ODE) for UV–vis measurements. The spectra are plotted in Figure 7A. A shoulder band started to rise at about 310 nm when the reaction mixture was heated to 100 °C. The band became more pronounced as the reaction temperature was increased to 215 °C and gradually evolved to a broad peak after the reaction had been allowed to proceed at 215 °C for 10 min. From this point, the intensity of the peak decreased with a further increased reaction time at 215 °C until the reaction was stopped at 120 min. We hypothesize that this peak could be associated with the electronic excitation of the Ni-TOP complexes present in the reaction. To verify this hypothesis, we used DFT to simulate the UV–vis spectrum of the complex. Figure 7B displays the TDDFT spectrum of a tetrahedral Ni(0)-TOP_4_ complex at the optimized geometry. The calculated spectrum shows a broad band at λ_max_ = 313 nm, which matches the peak position of the experimental spectra. Based on this finding, a reaction mechanism is proposed in Figure 7C, involving the intermediate Ni(0)-TOP_4_ complex. The reaction mechanism was further verified by reacting bis(1,5-cyclooctadiene)nickel(0) (Ni(COD)_2_) with TOP based on the hypothesis that the COD ligand would be replaced by TOP to form Ni(0)-TOP. The equivalent amount of Ni(COD)_2_, as that was used for Ni(acac)_2_ (0.2 mmol), was dispersed in 6 mL of TOP in a three-neck round-bottom flask under argon. The reaction mixture was heated to 215 °C and monitored by UV–vis spectroscopy. The absorbance peak at ∼313 nm that can be assigned to the Ni(0)-TOP_4_ complex appears as soon as Ni(COD)_2_ is mixed with TOP and becomes more obvious as the temperature increases to 215 °C (Figure S5). The UV–vis spectrum of Ni(COD)_2_, before TOP was introduced, shows a broad peak at 300 nm different from that of Ni(0)-TOP_4_. This experiment provides strong supporting evidence for our interpretation of UV–vis spectra. In addition, we calculated the UV–vis spectrum of Ni(II)-TOP_4_, which shows two peaks at 252 and 325 nm. This result also strongly supports that the single peak near 313 nm is characteristic of Ni(0). The composition of the product depends on the TOP/Ni(acac)_2_ ratio. At low TOP/Ni(acac)_2_ ratios, the product was dominated by metallic Ni, while uniform amorphous Ni_*x*_P_*y*_ nanoparticles were formed at high TOP/Ni(acac)_2_ ratios.

**Figure 8:**
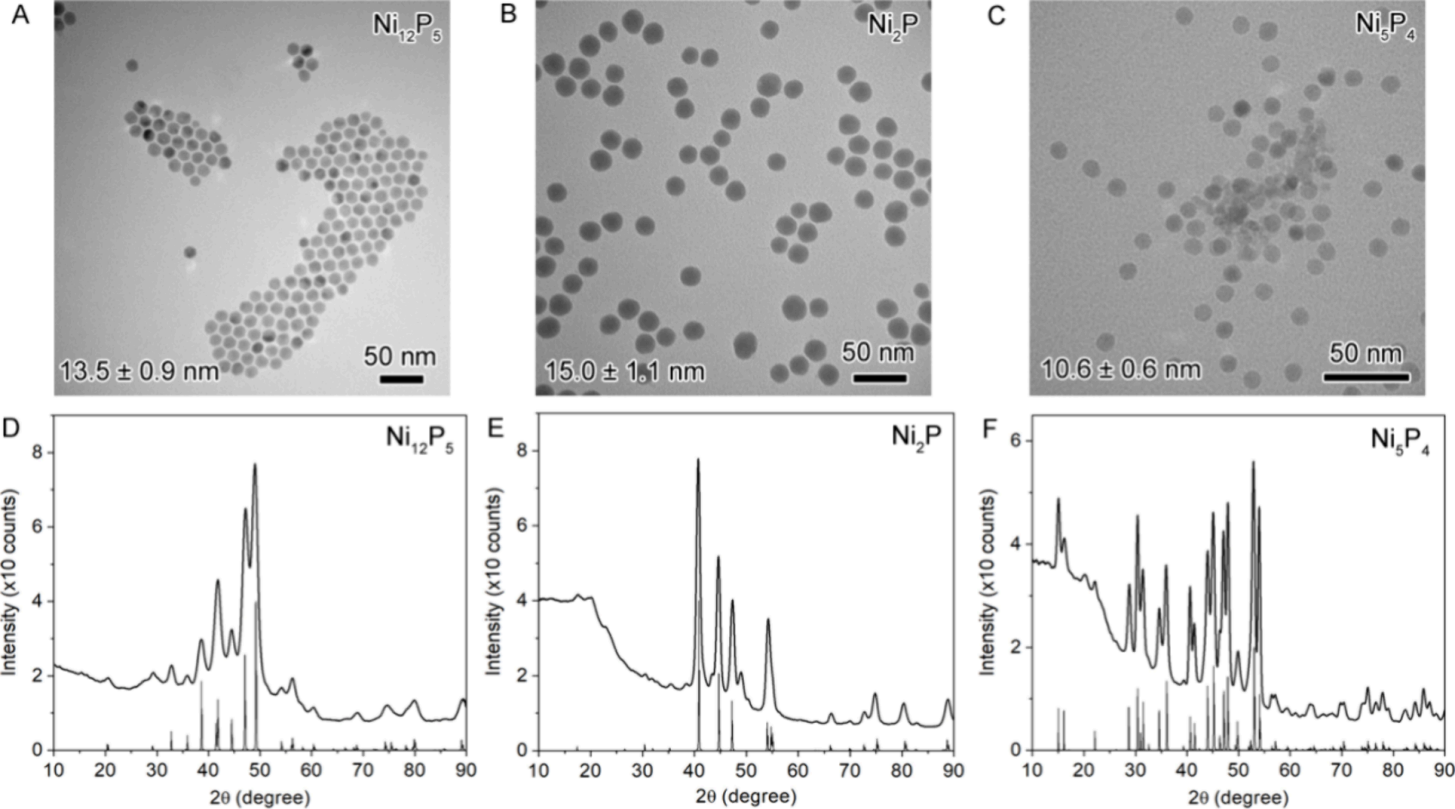
TEM and XRD characterization of phase-pure crystalline nanoparticles of Ni_12_P_5_, Ni_2_P, and Ni_5_P_4_: (A–C) TEM images and (D–F) XRD patterns.

Since the reaction with the TOP/Ni(acac)_2_ ratio of 11 yields uniform nanoparticles with a size on the order of 10 nm, we further investigated the effect of the temperature on the reaction. At 215 °C, the reaction yields amorphous Ni_*x*_P_*y*_ with an atomic Ni/P ratio of ∼75/25. Extending the reaction to 2 h, the composition slightly changes to Ni_70_P_30_ and the product remains amorphous. We hypothesize that elevating the temperature can promote the P incorporation and enhance crystallinity to access phase-pure crystalline nanoparticles with a similar size. To test the hypothesis, we carried out the reaction under the same conditions, except that after being kept at 215 °C for 1 h, the reaction was heated to a series of higher temperatures and annealed at that temperature for another hour. Through annealing at 280 °C, the reaction resulted in Ni_12_P_5_ nanoparticles, while at 315 and 350 °C, the reaction yielded Ni_2_P and Ni_5_P_4_ nanoparticles, respectively. As seen in the TEM images (Figure 8A–C), these crystalline nanoparticles have a similar size in the range of 10–15 nm. The XRD patterns in Figure 8D–F indicate that the samples are phase-pure, corresponding to Ni_12_P_5_,^45^ Ni_2_P,^32^ and Ni_5_P_4_.^46^

**Figure 9:**
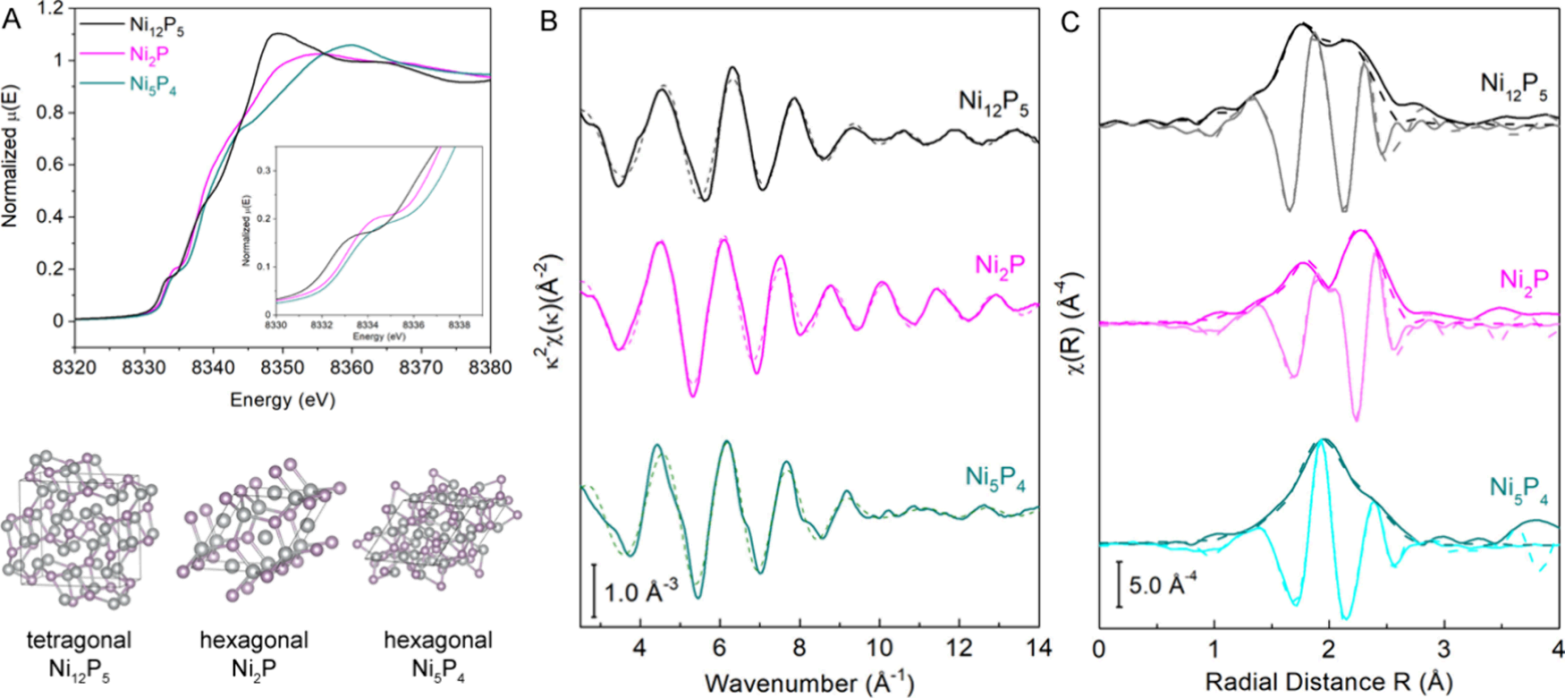
XAS characterization of phase-pure crystalline nanoparticles of Ni_12_P_5_, Ni_2_P, and Ni_5_P_4_: (A) Ni K-edge XANES spectra with the corresponding crystal structures; (B) Ni K-edge EXAFS data in K space with their corresponding fits (dashed lines); and (C) Ni K-edge EXAFS data in R space with the real (brightly colored) and imaginary (faded color) components for Ni_12_P_5_ (black), Ni_2_P (magenta), and Ni_5_P_4_ (cyan) with their corresponding fits (dashed lines).

We further characterized these phase-pure nanoparticles with a similar size by XAS. Figure 9A displays the Ni K-edge XANES comparison along with the crystal structures of the three phases—Ni_12_P_5_,^45^ Ni_2_P,^32^ and Ni_5_P_4_.^46^ Ni_12_P_5_ belongs to the tetragonal crystal system with two inequivalent Ni^1.25+^ ions, while Ni_2_P and Ni_5_P_4_ are associated with the hexagonal crystal system and contain a mixture of Ni^+^, Ni^2+^, and Ni^3+^. As XANES is sensitive to the oxidation state and coordination environment (e.g., geometry), the XANES region of these three phases is quite different from each other but are similar to those reported in the literature for the corresponding nanoscale Ni_12_P_5_,^47^ Ni_2_P,^48−52^ and Ni_5_P_4_.^50,53^ The pre-edge feature of Ni_12_P_5_ at 8333.0 eV slightly shifts down 1 eV from those of Ni_2_P and Ni_5_P_4_ at 8334.0 eV, probably due to a NiP_4_ tetrahedral geometry dominated in the Ni_12_P_5_ phase versus a NiP_5_ trigonal bipyramid dominated in Ni_2_P and Ni_5_P_4_ crystal structures. The EXAFS regions of these three nanoparticle samples were modeled using Ni–P and Ni–Ni scattering paths from their corresponding crystal structures Ni_12_P_5_,^45^ Ni_2_P,^32^ and Ni_5_P_4_.^46^ The fitting results are plotted in Figure 9B,C and are listed in Table S5. The Ni–P bond distances of all three nanoparticle samples (i.e., 2.21 ± 0.03, 2.24 ± 0.02, and 2.29 ± 0.01 Å for Ni_12_P_5_, Ni_2_P, and Ni_5_P_4_, respectively) are similar to those of their bulk structures (i.e., 2.22, 2.21, and 2.30 Å for Ni_12_P_5_, Ni_2_P, and Ni_5_P_4_, respectively). For the Ni–Ni bond length, the Ni_2_P nanoparticle sample has an average of 2.60 ± 0.01 Å similar to that of the bulk (2.61 Å), while Ni_12_P_5_ and Ni_5_P_4_ nanoparticle samples show 1.5 and 2.6% contractions compared to those of the bulk structures (2.50 ± 0.02 vs 2.54 Å for Ni_12_P_5_ and 2.58 ± 0.01 vs 2.65 Å for Ni_5_P_4_). The contraction is likely associated with the average size difference, as the size of the crystalline nanoparticles decreases in the order of Ni_2_P (15.0 ± 1.1 nm), Ni_12_P_5_ (13.5 ± 0.9 nm), and Ni_5_P_4_ (10.6 ± 0.6 nm).

## Conclusions

We have systematically studied the TOP-mediated synthesis of nickel phosphide nanoparticles, proposed a new reaction mechanism, and demonstrated a simple temperature-modulation means to synthesize phase-pure crystalline nanoparticles with a narrow size distribution. At a reaction temperature of 215 °C, the P incorporation into metallic Ni nanoparticles resulted in the formation of amorphous nickel phosphides. The reaction involved two competing processes: the addition of Ni(0) forming metallic Ni and the incorporation of P to the metallic Ni, yielding disordered nickel phosphides. The kinetics of these two processes depended on the ratio of TOP/Ni(acac)_2_ used in the synthesis. At low ratios (e.g., TOP/Ni(acac)_2_ = 0.5 and 2.5), the formation of metallic Ni dominated, while at high ratios (e.g., TOP/Ni(acac)_2_ = 11), uniform amorphous nickel phosphide nanoparticles were formed. A UV–vis study coupled with a DFT calculation verified the presence of Ni(0)-TOP_*x*_ complexes as intermediates in the synthesis. We further demonstrated the use of the highly uniform amorphous Ni_70_P_30_ nanoparticles as intermediates to obtain phase-pure Ni_12_P_5_, Ni_2_P, and Ni_5_P_4_ with narrow size distribution through the modulation of reaction temperature alone. This method, by transforming uniform amorphous to crystalline nanoparticles, may expand to the nanoparticle syntheses of other metal compounds.

## Supporting Information Available

ICP-MS results for the amorphous Ni_*x*_P_*y*_ nanoparticles; particle size measured from TEM; LCF analysis of amorphous Ni_*x*_P_*y*_ with different compositions; percentage composition of Ni and P derived from LCF; continuous Cauchy wavelet transform of a *k*^2^-weight in *R* space and *k*-range of 3.3–12; plots of the real and imaginary components of the Ni K-edge EXAFS results; EXAFS modeling results for the amorphous Ni_*x*_P_*y*_ and crystalline Ni_12_P_5_, Ni_2_P, and Ni_5_P_4_ nanoparticles; TEM, XRD, and ICP-MS of nanoparticles synthesized with TOP/Ni(acac)_2_ = 56 and 66; and UV–vis spectra of Ni(COD)_2_ in TOP at different temperatures and the calculated spectrum of Ni(II)-TOP_4_ (PDF)
