# Supplementary material for: Synthesis of Amorphous and Various Phase-Pure Nanoparticles of Nickel Phosphide with Uniform Sizes via a Trioctylphosphine-Mediated Pathway
Source: Inorg Chem. 2024 Sep 27;63(40):18981–91. doi: 10.1021/acs.inorgchem.4c03334 (PMC11462502; doi:10.1021/acs.inorgchem.4c03334)
Supplement: Supplementary file 1 — ic4c03334_si_001.pdf [file ic4c03334_si_001.pdf]

Supporting Information

Synthesis of Amorphous and Various Phase-Pure Nanoparticles of Nickel Phosphide with Uniform Sizes *via* a Trioctylphosphine-Mediated Pathway

David Thompson[a], Adam S. Hoffman[b], Zachary R. Mansley[c], Sarah York[a], Feng Wang[a], Yimei Zhu[d], Simon R. Bare[b], and Jingyi Chen[a*]

[a]*Department of Chemistry and Biochemistry, University of Arkansas, Fayetteville, AR 72701, United States*

[b]*Stanford Synchrotron Radiation Lightsource, SLAC National Accelerator Laboratory, Menlo Park, CA 94025, United States*

[c]*Interdisciplinary Science Department, Brookhaven National Laboratory, Upton, NY 11973, United States*

[d]*Condensed Matter Physics and Materials Science Department, Brookhaven National Laboratory, Upton, NY 11973, United States*

[*]Email: chenj@uark.edu

Table S1. Summary of ICP-MS results for the amorphous Ni_xP_y nanoparticles synthesized under different conditions by varying the amount of TOP used and the reaction time.

Amount of TOP (mL/mmol)	Reaction time (min)	Ni (mol%)	P (mol%)
0.05/0.11	10	-	-
	30	72	28
	60	99	1
	120	98	2
0.20/0.45	10	98	2
	30	98	2
	60	95	5
	120	96	4
0.50/1.12	10	94 ± 1	6 ± 1
	30	87 ± 8	13 ± 8
	60	86 ± 10	14 ± 10
	120	74 ± 3	26 ± 3
1.00/2.24	10	77 ± 6	23 ± 6
	30	75 ± 4	25 ± 4
	60	72 ± 3	28 ± 7
	120	73 ± 3	27 ± 7

Table S2. Summary of the particle size measured from TEM and the formula derived from ICP-MS results for each sample.

Amount of TOP (mL/mmol)	Reaction time (min)	Size from TEM (nm)	Formula derived from ICP-MS
0.05/0.11	10	-	-
	30	7.7 ± 1.0	$Ni_{72}P_{28}$
	60	48.4 ± 8.3	$Ni_{99}P_{1}$
	120	52.8 ± 8.8	$Ni_{98}P_{2}$
0.20/0.45	10	31.6 ± 3.7	$Ni_{98}P_{2}$
	30	29.3 ± 2.9	$Ni_{98}P_{2}$
	60	33.7 ± 5.4	$Ni_{95}P_{5}$
	120	39.8 ± 3.9	$Ni_{96}P_{4}$
0.50/1.12	10	21.8 ± 2.5	$Ni_{94}P_{6}$
	30	21.0 ± 3.3	$Ni_{87}P_{13}$
	60	20.4 ± 3.8	$Ni_{86}P_{14}$
	120	12.0 ± 1.0	$Ni_{76}P_{24}$
1.00/2.24	10	10.0 ± 1.0	$Ni_{74}P_{26}$
	30	12.5 ± 1.0	$Ni_{72}P_{28}$
	60	12.7 ± 1.0	$Ni_{72}P_{28}$
	120	15.7 ± 2.0	$Ni_{75}P_{25}$

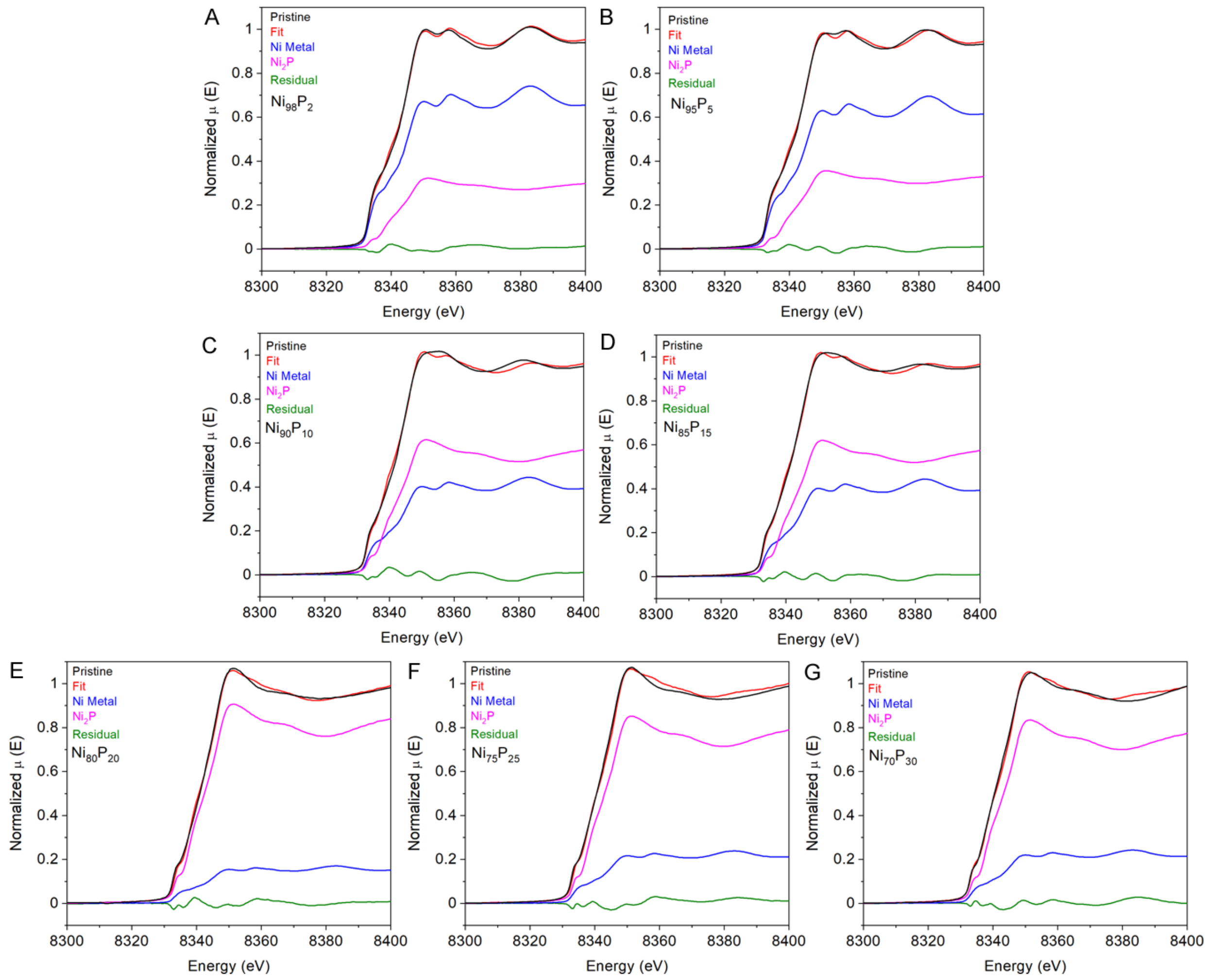

Figure S1. LCF analysis of amorphous Ni_xP_y with different compositions using the XAS of metallic Ni and Ni_2P data: (A) $Ni_{98}P_2$; (B) $Ni_{95}P_5$; (C) $Ni_{90}P_{10}$; (D) $Ni_{85}P_{15}$; (E) $Ni_{80}P_{20}$; (F) $Ni_{75}P_{25}$; and (G) $Ni_{70}P_{30}$.

Table S3. Summary of the percentages of Ni and P derived from the LCF analysis of the XANES data of the amorphous Ni_xP_y nanoparticles.

Ni_xP_y	Ni metal (%) from LCF	Ni_2P (%) from LCF	Ni (%)[a]	P (%)[b]
$Ni_{98}P_2$	71.5 ± 1.3	30.0 ± 1.2	91.5	8.5
$Ni_{95}P_5$	67.0 ± 1.3	33.2 ± 1.2	89.1	10.9
$Ni_{90}P_{10}$	42.8 ± 1.9	57.3 ± 1.8	81.0	19.0
$Ni_{85}P_{15}$	42.8 ± 1.4	57.8 ± 1.3	81.3	18.7
$Ni_{80}P_{20}$	16.4 ± 1.8	84.5 ± 1.6	72.8	27.2
$Ni_{75}P_{25}$	23.0 ± 2.1	79.4 ± 2.0	76.0	24.0
$Ni_{70}P_{30}$	23.4 ± 1.6	77.8 ± 1.5	75.3	24.7

[a]: The percentage of Ni is calculated from the LCF results using equation.

$$\text{Ni (\%)} = \text{Ni metal (\%)} + \text{Ni}_2\text{P (\%)} \times 2/3$$

[b]: The percentage of P is calculated using equation: P (%) = 100 – Ni (%).

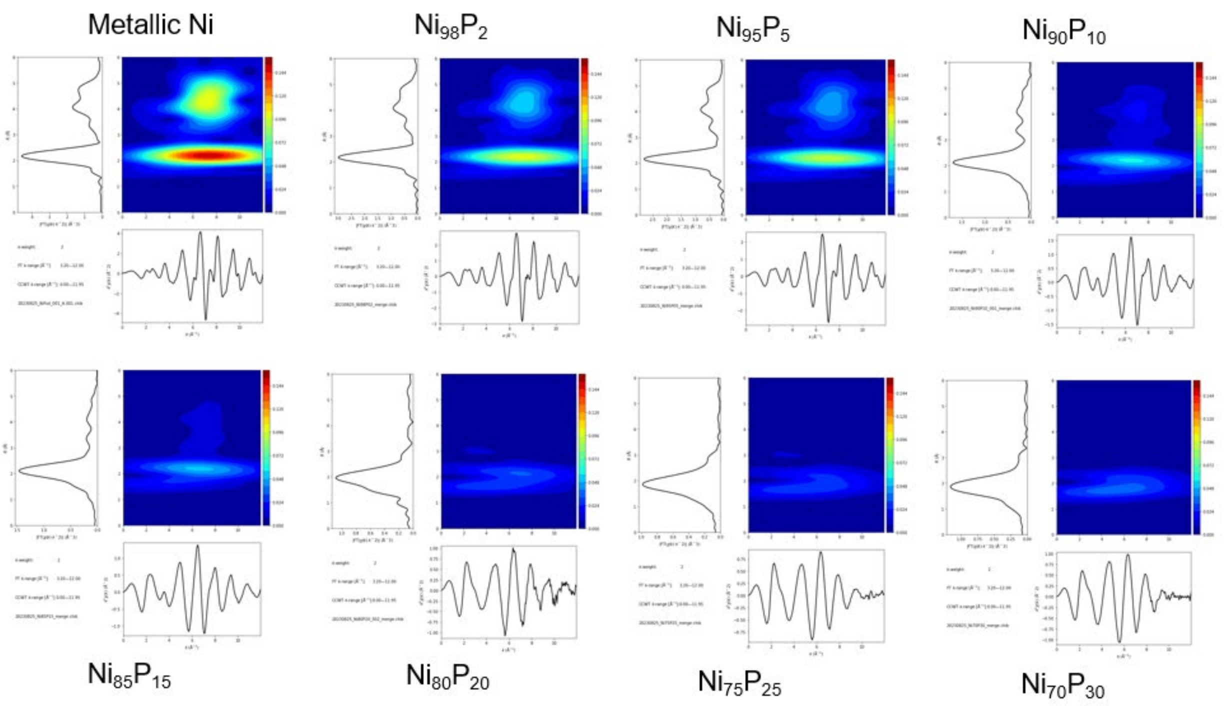

Figure S2. Continuous cauchy wavelet transform of a k^2-weight in *R* space and *k*-range of 3.3-12 for the series of samples from metallic Ni to amorphous Ni_xP_y with different compositions. The intensity scale is the same for each data set.

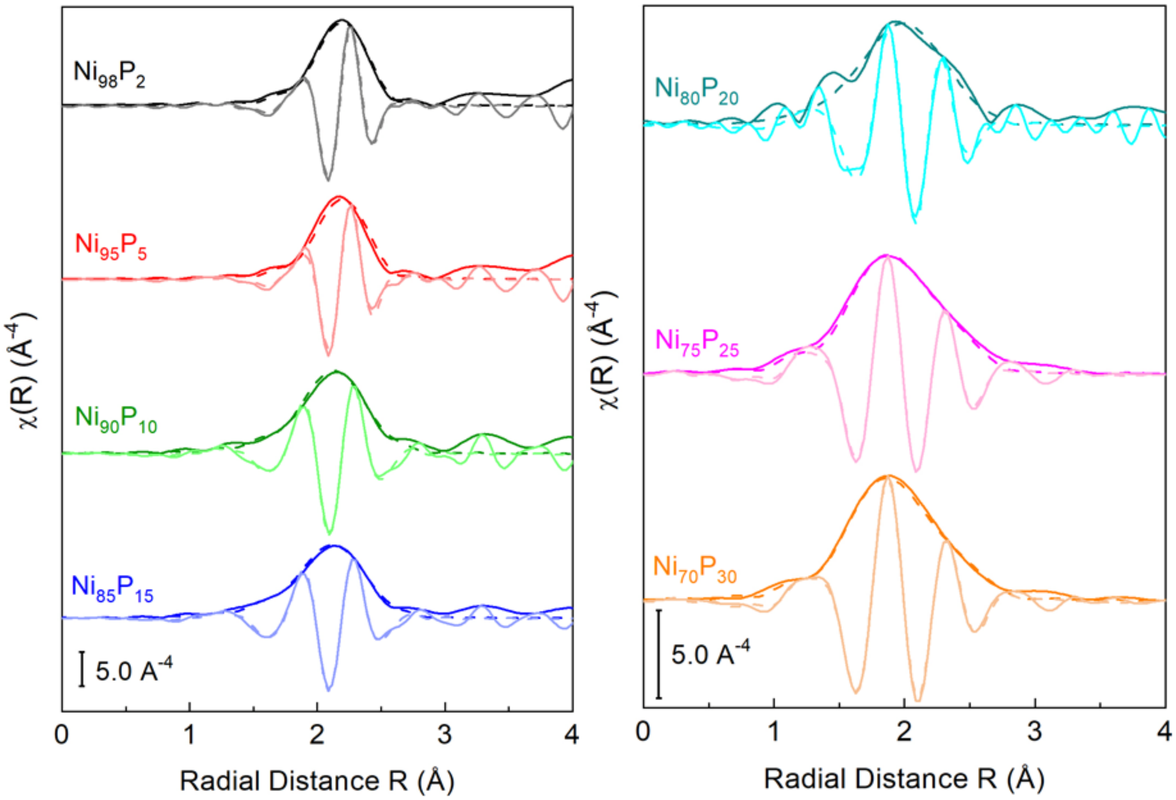

Figure S3. The real (brightly colored) and imaginary (faded color) components of the Ni K-edge EXAFS results for $Ni_{98}P_2$ (black), $Ni_{95}P_5$ (red), $Ni_{90}P_{10}$ (green), $Ni_{85}P_{15}$ (blue), $Ni_{80}P_{20}$ (cyan), $Ni_{75}P_{25}$ (magenta), $Ni_{70}P_{30}$ (orange) with their corresponding fits (dashed lines).

Table S4. Ni K-edge EXAFS fit results for Ni_xP_y where x and y were the mol% of each sample derived from the ICP-MS results.

Sample	Structure	Scattering Path	R(Å)	CN	σ^2	E_o (eV)	Reduced χ^2	R-factor
Metallic Ni (Ref.)		*Ni-Ni*	2.49	12				
Ni_2P (Ref.)		*Ni-P*	2.21	2				
		Ni-Ni	2.61	4				
$Ni_{98}P_{02}$	*fcc* Ni	*Ni-Ni*	2.49±0.00	9.3±0.6	0.007±0.000	6.75±0.45	123.2	0.015
TOP/Ni(acac)$_2$: 0.5								
Rxn. time: 120 min								
$Ni_{98}P_{02}$	fcc Ni	*Ni-Ni*	2.48±0.01	9.1±0.5	0.006±0.000	7.5±0.23	66014	0.011
	Ni_2P	*Ni-Ni*	2.60±0.01	1.5±0.6	0.005±0.000	-2.45±0.83		
	Ni_2P	*Ni-P$_1$*	2.21±0.00	0.4±0.3	0.007±0.001	-2.45±0.83		
$Ni_{95}P_{05}$	*fcc* Ni	*Ni-Ni*	2.49±0.00	8.7±0.5	0.007±0.000	6.75±0.45	123.2	0.015
TOP/Ni(acac)$_2$: 2.5								
Rxn. time: 60 min								
$Ni_{95}P_{05}$	fcc Ni	*Ni-Ni*	2.48±0.01	9.8±0.5	0.006±0.000	7.5±0.23	66014	0.011
	Ni_2P	*Ni-Ni*	2.60±0.01	3.2±0.6	0.005±0.000	-2.45±0.83		
	Ni_2P	*Ni-P$_1$*	2.21±0.00	0.3±0.2	0.007±0.001	-2.45±0.83		
$Ni_{90}P_{10}$	*fcc* Ni	*Ni-Ni*	2.49±0.00	7.4±0.6	0.007±0.000	6.75±0.45	123.2	0.015
TOP/Ni(acac)$_2$: 5.5	Ni_2P	*Ni-Ni*	2.61±0.01	3.2±0.6	0.006±0.001	-0.46±0.97		0.015
Rxn. time: 10 min	Ni_2P	*Ni-P$_1$*	2.23±0.01	0.9±0.3	0.008±0.002	-0.46±0.97		0.015
$Ni_{85}P_{15}$	*fcc* Ni	*Ni-Ni*	2.49±0.00	6.2±0.4	0.007±0.000	6.75±0.45	123.2	0.015
TOP/Ni(acac)$_2$: 5.5	Ni_2P	*Ni-Ni*	2.61±0.01	2.5±0.5	0.006±0.001	-0.46±0.97		0.015
Rxn. time: 30 min	Ni_2P	*Ni-P$_1$*	2.23±0.01	1.2±0.2	0.008±0.002	-0.46±0.97		0.015
$Ni_{80}P_{20}$	*fcc* Ni	*Ni-Ni*	2.49±0.00	3.5±1.1	0.007±0.000	6.75±0.45	123.2	0.015
TOP/Ni(acac)$_2$: 5.5	Ni_2P	*Ni-Ni*	2.61±0.01	1.4±1.1	0.006±0.001	-0.46±0.97		0.015
Rxn. time: 120 min	Ni_2P	*Ni-P$_1$*	2.23±0.01	2.4±0.8	0.008±0.002	-0.46±0.97		0.015
$Ni_{75}P_{25}$	*fcc* Ni	*Ni-Ni*	2.49±0.00	2.9±0.3	0.007±0.000	6.75±0.45	123.2	0.015
TOP/Ni(acac)$_2$: 11	Ni_2P	*Ni-Ni*	2.61±0.01	1.8±0.3	0.006±0.001	-0.46±0.97		0.015
Rxn. time: 10 min	Ni_2P	*Ni-P$_1$*	2.23±0.01	2.5±0.3	0.008±0.002	-0.46±0.97		0.015
$Ni_{70}P_{30}$	*fcc* Ni	*Ni-Ni*	2.49±0.00	3.3±0.5	0.007±0.000	6.75±0.45	123.2	0.015
TOP/Ni(acac)$_2$: 11	Ni_2P	*Ni-Ni*	2.61±0.01	2.4±0.5	0.006±0.001	-0.46±0.97		0.015
Rxn. time: 60 min	Ni_2P	*Ni-P$_1$*	2.23±0.01	3.0±0.3	0.008±0.002	-0.46±0.97		0.015

Table S5. Ni K-edge EXAFS fit results for crystalline nickel phosphide nanoparticles and their comparison to the bulk crystalline structures.

Sample	Structure	Scattering Path	R(Å)	CN	σ^2	E_o (eV)	Reduced χ^2	R-factor
$Ni_{12}P_5$ (ICSD# 108640)		*Ni-P*	2.22	1				
		Ni-Ni	2.54	3				
$Ni_{12}P_5$ nanoparticles	$Ni_{12}P_5$	*Ni-P*	2.21±0.03	2.6±0.06	0.006±0.003	-4.1±3.2	6549.3	0.038
		Ni-Ni	2.50±0.02	1.6±0.09	0.005±0.003	-4.1±3.2	6549.3	0.038
Ni_2P (ICSD# 27162)		*Ni-P*	2.21	2				
		Ni-Ni	2.61	4				
Ni_2P nanoparticles	Ni_2P	*Ni-P*	2.24±0.02	2.2±0.5	0.007±0.002	-2.9±1.8	7056.4	0.013
		Ni-Ni	2.60±0.01	3.1±0.7	0.006±0.001	-2.9±1.8	7056.4	0.013
Ni_5P_4 (ISCD# 76671)		*Ni-P*	2.30	2				
		Ni-Ni	2.65	4				
Ni_5P_4 nanoparticles	Ni_5P_4	*Ni-P*	2.29±0.01	3.8±0.7	0.006±0.001	1.9±1.4	3270.2	0.006
		Ni-Ni	2.58±0.01	5.2±1.6	0.001±0.003	1.9±1.4	3270.2	0.006

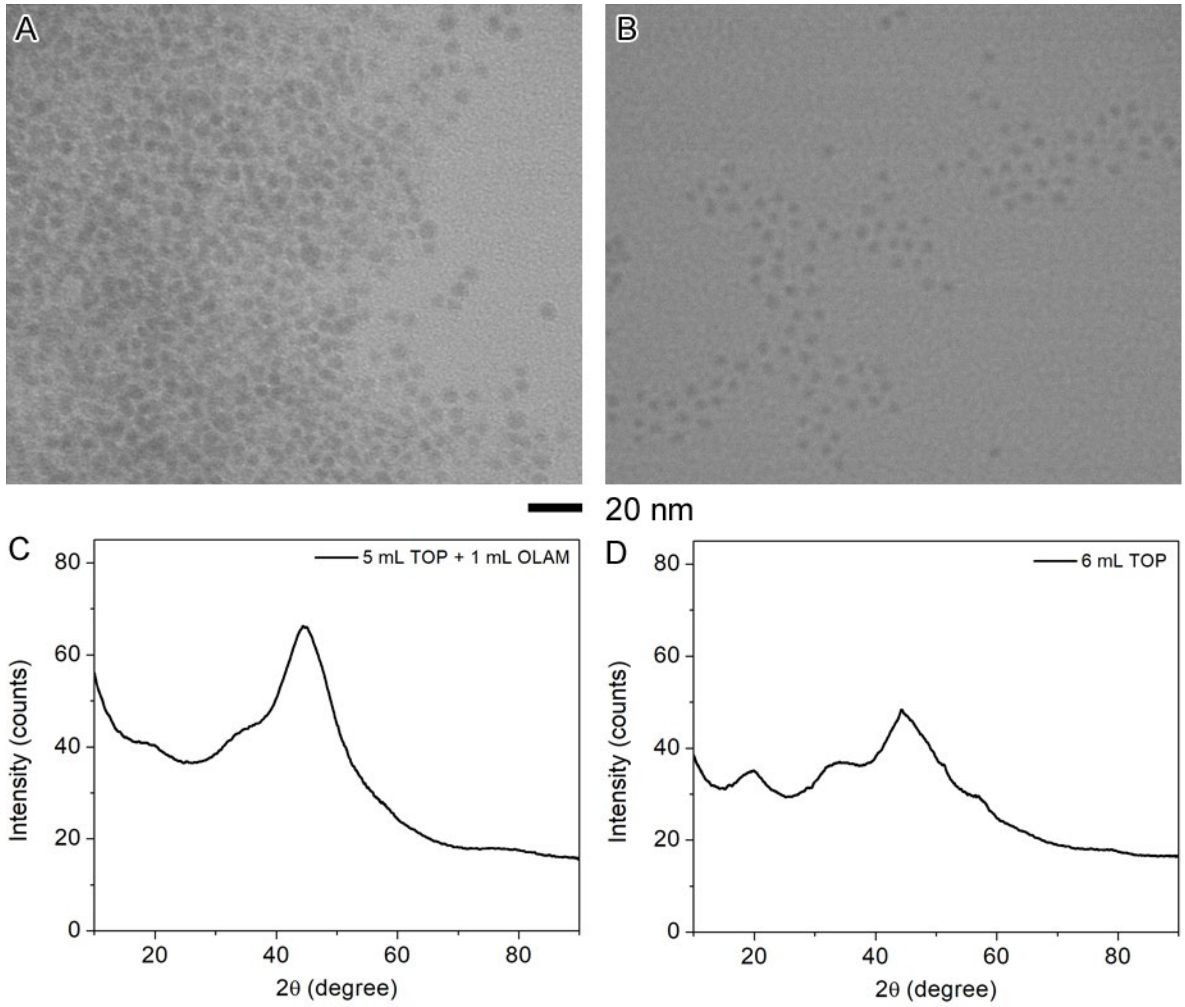

Figure S4. (A) TEM image of $Ni_{69}P_{31}$ nanoparticles (5.0 ± 1.0 nm) synthesized at TOP/Ni(acac)$_2$ = 56 in 5 mL of TOP and 1 mL of OLAM; (B) TEM image of $Ni_{69}P_{31}$ nanoparticles (3.8 ± 0.6 nm) synthesized at TOP/Ni(acac)$_2$ = 66 in 6 mL of TOP alone; (C) XRD pattern of the sample in (A); and (D) XRD pattern of the sample in (B). The elemental compositions of the samples were measured by ICP-MS.

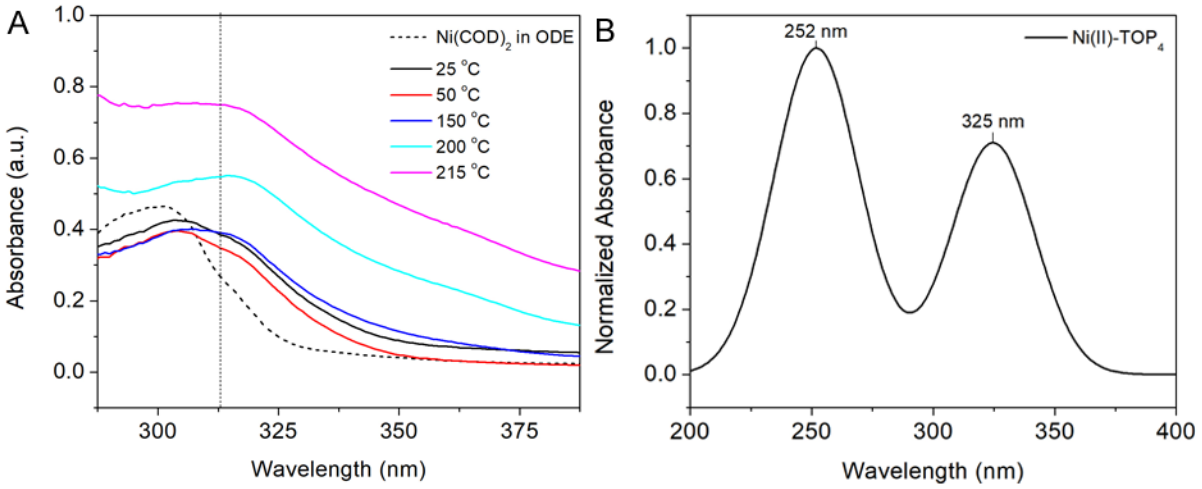

Figure S5. (A) UV-vis spectra of the aliquots taken from the reaction where 40 mg bis(1,5-cyclooctadiene)nickel(0) ($Ni(COD)_2$) was reacted with 6 mL of TOP under argon. For comparison, the UV-vis spectrum of $Ni(COD)_2$ (λ_{max} = 300 nm) before TOP was introduced is also provided (dashed line). (B) Calculated UV-vis spectrum of a tetrahedral Ni(II)-TOP_4 complex showing two peaks at 252 and 325 nm, respectively.
